# Recombinase Polymerase Amplification Combined with Fluorescence Immunochromatography Assay for On-Site and Ultrasensitive Detection of SARS-CoV-2

**DOI:** 10.3390/pathogens11111252

**Published:** 2022-10-28

**Authors:** Guangyu Wang, Xingsheng Yang, Hao Dong, Zhijie Tu, Yong Zhou, Zhen Rong, Shengqi Wang

**Affiliations:** 1 Beijing Institute of Microbiology and Epidemiology, Beijing 100850, China; 2 National Institutes for Food and Drug Control, Beijing 100050, China; 3 University of Science and Technology of China, Hefei 230036, China

**Keywords:** immunochromatographic assay, recombinase polymerase amplification, POCT, SARS-CoV-2, multilayer quantum dot

## Abstract

This study established a portable and ultrasensitive detection method based on recombinase polymerase amplification (RPA) combined with high-sensitivity multilayer quantum dot (MQD)-based immunochromatographic assay (ICA) to detect the severe acute respiratory syndrome coronavirus 2 (SARS-CoV-2). The RPA-MQD-based ICA method is reported for the first time and has the following advantages: (i) RPA is free from the constraints of instruments and can be promoted in point-of-care testing (POCT) scenarios, (ii) fluorescence ICA enhances the portability of detection operation so that the entire operation time is controlled within 1 h, and (iii) compared with common colorimetric-based RPA-ICA, the proposed assay used MQD to provide strong and quantifiable fluorescence signal, thus enhancing the detection sensitivity. With this strategy, the proposed RPA-MQD-based ICA can amplify and detect the SARS-CoV-2 nucleic acid on-site with a sensitivity of 2 copies/reaction, which is comparable to the sensitivity of commercial reverse transcription quantitative polymerase chain reaction (RT-qPCR) kits. Moreover, the designed primers did not cross-react with other common respiratory viruses, including adenovirus, influenza virus A, and influenza virus B, suggesting high specificity. Thus, the established portable method can sensitively detect SARS-CoV-2 nucleic acid without relying on equipment, having good application prospects in SARS-CoV-2 detection scenarios under non-lab conditions.

## 1. Introduction

Severe acute respiratory syndrome coronavirus 2 (SARS-CoV-2) is the causative agent of the coronavirus disease 2019 (COVID-19) pandemic [1,2,3,4], which has led to more than 605 million infections and 6.5 million deaths worldwide (https://covid19.who.int/; accessed on 5 September 2022). At present, direct virus detection methods are based on the sequence specificity of viral nucleic acid and the epitope specificity of viral capsid protein [5,6]. Due to the high sensitivity and specificity, nucleic acid-based reverse transcription quantitative polymerase chain reaction (RT-qPCR) is considered the gold standard approach [7,8]. RT-qPCR is based on the principle of complementary base pairing of nucleic acids and achieves exponential amplification of target fragments under the action of DNA polymerase. As the amplification reaction proceeds, the fluorescence signal gradually accumulates and can be read by the detector when the detection threshold is reached. Base pairing ensures the specificity, and exponential amplification and sensitive fluorescence signal ensure the sensitivity of the reaction. However, PCR-based methods still require a clean environment, large-scale instruments and professional technicians, thereby limiting their application in point-of-care testing (POCT) areas [9].

For protein-based immunochromatographic techniques, specific antibodies to the viral capsid must be prepared in advance. When the virus binds to the antibody, it produces a signal visible to the naked eye. This method is easy to operate, does not depend on large equipment, and can achieve the purpose of the rapid inspection; however, it also has low sensitivity [10,11,12,13]. However, recent studies have shown that the sensitivity of detection can be significantly improved by using new materials such as fluorescence nanotags, magnetic particles and surface-enhanced Raman scattering (SERS) sensors [14,15,16]. The most representative quantum dot fluorescent materials have obvious characteristics, including high photostability, narrow fluorescence emission spectrum, and strong luminescence. Therefore, quantum dot nanoprobes can provide strong and quantifiable fluorescent signals for immunochromatographic assay (ICA). Furthermore, quantum dot beads can be fabricated by encapsulating many quantum dots into polymers or silica micelles, which can provide high luminescence and stability [17,18,19,20,21,22]. Many previous studies have demonstrated that the combination of quantum dot beads and ICA strips can greatly improve the sensitivity of the common colloidal gold (AuNP)-ICA method [23,24,25,26,27]. For example, Wang et al. developed a QD-based ICA for rapid detection of SARS-CoV-2 nucleocapsid protein (NP) with the limit of detection (LOD) reaching 5 pg/mL, which is 100 times more sensitive than the AuNP-ICA based method [28]. This work strongly illustrates that QD fluorescent material can significantly enhance the detection sensitivity of ICA for the virus. However, for ICA-based antigen detection assay, there are several deficiencies that need to be addressed, such as a high false negative rate, missed detection of variants, and usually need a pair of specific antibodies. Therefore, it is difficult for this method to completely replace nucleic acid detection.

The recombinase polymerase amplification (RPA) technology developed in recent years can search for homologous sequences in double-stranded DNA templates by combining recombinase with primers to form protein-DNA complexes. Once the primers locate the homologous sequence, a strand-exchange reaction occurs. Then, the target region on the template exponentially amplifies. Because the principle of nucleic acid amplification is different from that of traditional PCR, the RPA reaction temperature is a constant temperature of 37 °C. Thus, RPA has very low requirements on technology and equipment. By combining with some ICA, it is especially suitable for portable detection [29,30,31,32]. A previous study has shown that SARS-CoV-2 detection sensitivity of RPA combined with colloidal gold technology can reach 30 copies/reaction [33]. In theory, with more sensitive multilayer quantum dot fluorescent materials, the sensitivity can be further improved.

Herein, we established a portable and ultrasensitive detection method based on RPA combined with high-sensitivity multilayer quantum dot-based immunochromatography to detect SARS-CoV-2. Nucleic acid amplification methods and fluorescent materials were used to improve sensitivity, and an RPA isothermal amplification system combined with immunochromatography was adopted to guarantee portability. The detection process is divided into two stages: nucleic acid amplification and immunodetection.

In the RPA amplification process, the RNA template is first reverse transcribed into cDNA under the action of reverse transcriptase. Then, the target fragment is amplified using recombinases, single-stranded DNA binding proteins, DNA polymerases, and labeled primers. The temperature of the reaction system was 37 °C, and the reaction time was 30 min. Comparatively, each cycle of conventional PCR requires a temperature change process, which not only requires the participation of the instrument but also consumes time during heating and cooling. Apart from that, the amplification procedure takes at least 1 h. The RPA reaction can always be maintained at about 37 °C, and the reaction time is 15–30 min. The characteristics of constant temperature and rapid response make RPA fully applicable to rapid on-site inspection.

In the ICA process, the RPA product with biotin and digoxin was first attached to the quantum dot-labeled digoxin antibody. Then, the RPA product-antibody complex flowed along the strip due to capillary action. When the biotin at the other end of the RPA product is combined with SA, immuno-SiO_2_-MQD stays at the T line, and the unbound labeled antibody continues to move until the goat anti-mouse antibody at the C line binds. Approximately 15 min later, a bright red T line can be seen under ultraviolet light ICA, and the result is recorded by a mobile phone camera.

The advantages of the study could be summarized in three points: (1) the amplification method adopts RPA instead of ordinary PCR, which is free from the constraints of instruments and can be promoted in POCT scenarios; (2) the use of ICA enhances the portability of the operation so that the entire operation time is controlled within 1 h; (3) nucleic acid amplification combined with multilayer quantum dot nanoparticles (MQDs) enhances the detection sensitivity. The established scheme may have wide applications in limited primary hospitals, underdeveloped areas, and self-examination at home.

## 2. Experimental Section

### 2.1. Reagents and Materials

RoomTemp Sample Lysis Kit was obtained from Vazyme Biotech (Nanjing, China). ProtoScript II RT and Rnase H were supplied by NEB (Beijing, China). TwistAmp^®^ Basic Kit was purchased from TwistDx (Cambridge, UK). Primers were synthesized, modified, and purified by General Biotech (Hefei, China). Streptavidin and digoxin antibodies were acquired from Biocare Biotech (Zhuhai, China) and Meridianlifescience (Beijing, China). The goat anti-mouse IgG antibody was purchased from Sigma (Shanghai, China). Inactivated Adenovirus (ADV, ~10^9^ copies/μL) was retained by our laboratory and inactivated. Influenza A virus (IAV, ~10^5^ copies/μL), influenza B virus (IBV, ~10^8^ copies/μL), and SARS-CoV-2 virus (~10^8^ copies/μL) were a kind gift from the Respiratory Virus Vaccine department of the National Institute for Food and Drug Control (Beijing, China).

Branched polyethyleneimine (PEI, MW ~25 kDa), tetraethyl orthosilicate (TEOS), N-hydroxy-sulfosuccinimide (sulfo-NHS), 2-(N-morpholino) ethanesulfonic (MES), N-(3-dimethylaminopropyl)-N′ethylcarbodiimide hydrochloride (EDC), and CdSe/ZnS-COOH QDs (excitation/emission maxima ~365/625 nm) were supplied by Mesolight Inc. (Suzhou, China). UniSart CN140 nitrocellulose (NC) membrane was provided by Sartorius (Göttingen, Germany). Other ICA materials, including a sample pad, polyvinyl chloride bottom plate, and absorption pad, were supplied by Jieyi Biotechnology Co. (Shanghai, China).

### 2.2. Nucleic Acid Release

In accordance with the instruction manual, viral nucleic acid templates were released by the RoomTemp Sample Lysis Kit for 10 min at room temperature. Virus lysates and dilutions were used in subsequent experiments.

### 2.3. Primer Design

We designed three pairs of RPA primers. Two pairs of primers were selected from the reference [34]. By verifying the above 5 pairs of primers one by one, only one pair of primers was screened to amplify SARS-CoV-2 without non-specific amplification. The amplified product (201 bp) is a part of the S protein gene. The optimal primers are as follows:

5′-Digoxin-CCACTGAGAAGTCTAACATAATAAGAGGCTG-3′, 5-Biotin-AATAAACTCTGAACTCACTTTCCATCCAACT-3′

### 2.4. RT-RPA Assay

The reverse transcription RPA (RT-RPA) system contains pre-packaged and lyophilized enzyme mixture, 29.5 μL of Primer Free Rehydration buffer, 1 μL of each optimized primer (2.5 μM) for SARS-CoV-2, 2 μL of RNA template for SARS-CoV-2, 0.5 μL of ProtoScript II RT, 1 μL of Rnase H, 12.5 μL of double-distilled water and 2.5 μL of 280 mM magnesium acetate in a total volume of 50 μL. The nucleic acid amplification with a water bath at 37 °C was completed in 30 min.

### 2.5. Synthesis of SiO_2_-MQDs Nanocomposite

Firstly, the synthesis of SiO_2_ NPs was carried out according to the method previously published by our laboratory [35]. Subsequently, the surface of SiO_2_ NPs was labeled with quantum dots by PEI-mediated electrostatic adsorption. The preparation process was as follows: (1) to prepare PEI-coated SiO_2_ NPs (SiO_2_/PEI), 1 mL of SiO_2_ NPs (1 mg/mL) and 40 mL of aqueous PEI solution (0.2 mg/mL) were mixed and sonicated vigorously for one hour at room temperature; (2) after centrifugation (6000 rpm, 6 min), PEI-coated SiO_2_ NPs were washed with deionized water three times; (3) the SiO_2_/PEI NPs were mixed and reacted with 40 mL of CdSe/ZnS-MPA QDs (1 nM) under ultrasonic conditions for 60 min at room temperature. Transmission electron microscopy (TEM) was used to evaluate whether quantum dots are absorbed on the surface of the SiO_2_ NPs. After centrifugation (5400 rpm, 6 min) and washing, the prepared SiO_2_-QDs were resuspended in 1 mL of deionized water. The above steps were repeated twice to prepare SiO_2_-MQDs with high fluorescence intensity encapsulated by two-layer quantum dots (Dutta et al., 2022 [7]).

### 2.6. Preparation of Immuno-SiO_2_-MQDs Labels

Digoxin monoclonal antibody was conjugated with SiO_2_-MQDs via carbodiimide chemistry at room temperature [36]. First, 1 mL of SiO_2_-MQD was mixed with MES solution (0.1 M) with 1 mM EDC and 2 mM sulfo-NHS and then sonicated for 15 min. After centrifugation (4200 rpm, 6 min) and resuspension in 0.2 mL of PBS, the mixture was reacted with 15 μg of digoxin monoclonal antibody for two hours, followed by surface blocking with BSA for another one hour. Finally, immuno-SiO_2_-MQD labels were centrifuged (4200 rpm, 6 min), washed twice with PBS, redispersed in 1 mL of PBS solution, and stored at 4 °C.

### 2.7. Preparation of the ICA Strip

The ICA strip consisted of a sample pad, an NC membrane with one test line (T line), one control line (C line), and an absorbent pad. Streptavidin (1.0 mg/mL) and goat-anti-mouse IgG antibody (1.0 mg/mL) were spotted on the NC membrane to form the T line and the C line with a constant dispense of 0.1 μL/mm by the XYZ spraying platform (Biodot, Irvine, CA, USA) [37]. All the ICA components were placed into a drying oven at 37 °C for 2 h and then attached to the plastic backing card in sequence. Eventually, the assembled ICA card was cut into 3 mm-wide strips and preserved in a desiccator until use.

### 2.8. Analytical Procedure for Detection of SARS-CoV-2

An ICA strip was used to test the RPA products. First, RPA products (2 μL) were mixed with 2 μL immuno-SiO_2_-MQD labels and 70 μL running buffer solution (10 mM PBS, pH 7.4, 0.05% Tween-20, 1% BSA). Then, a sample pad of ICA strip was immersed into the mixture. Fifteen minutes later, the T line turned into a bright red line under the Handheld UV light (365 nm) illumination (Shenyu, Dongying, China) in the presence of the RPA products. Results could be read by the naked eye or photographed with a mobile phone.

### 2.9. Optimization of Experimental Procedures and Methodological Investigation

Considering that the reaction temperature of reverse transcription and RPA is similar, we tried to combine the two steps into one. In addition, the primer concentration and the amount of product detection were optimized. For methodological validation, we examined the specificity, sensitivity and reproducibility of the established method.

## 3. Results and Discussion

### 3.1. Construction of Immuno-MQD Labels and MQD-Based ICA

**Scheme 1:**
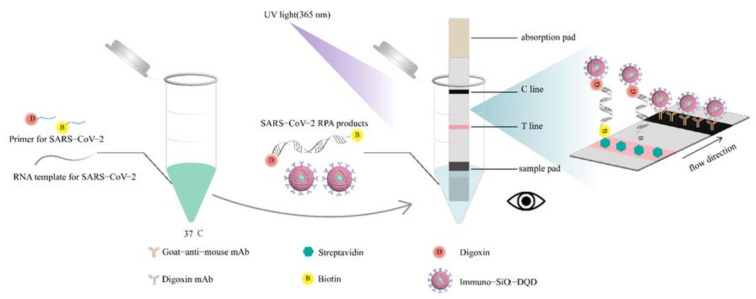
Schematic diagram of RT-RPA and operating principle of SiO_2_-MQD-ICA strip for detecting RPA products.

**Figure 1:**
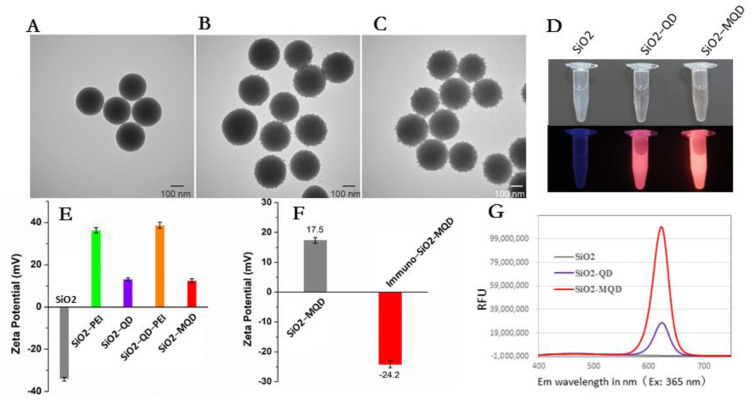
Characterization of the fabricated SiO_2_—MQD nanocomposite. TEM images of single SiO_2_ core (**A**), SiO_2_—QD (**B**), SiO_2_—MQD (**C**), and the fluorescence emission spectra of synthesized NPs from each stage (**D**). The inset in (**D**) displays the photographs of these NPs solutions under visible (upper) and UV light (lower). Zeta potentials (**E**,**F**) and fluorescence emission spectra (**G**) of the synthesized NPs from each stage.

Herein, a high-performance MQD was prepared through the layer-by-layer assembly of highly photostable CdSe@ZnS-COOH QDs onto the surface of monodispersed SiO_2_ NP, which can provide higher QD loads, better luminescence, good stability and dispersibility than traditional spherical fluorescent microspheres for ICA methods (Scheme 1 and Figure 1A–C). As observed in Figure 1D–G, this preparation method increases the fluorescence of the material. Moreover, the two-layer shell of SiO_2_-MQD increases the surface area of SiO_2_ microspheres and provides more carboxyl groups, which is more conducive to the covalent attachment of antibodies [7]. As revealed in Figure 1E, the zeta potential of SiO_2_-based nanocomposites increased significantly after PEI combining and decreased sharply after QDs coating. The zeta potential values are 33.7, 36.6, 12.9, 38.9, and 12.3 mV for SiO_2_, SiO_2_-PEI, SiO_2_-QD, SiO_2_-QD-PEI and SiO_2_-MQD, respectively. Such a high and low potential change proves that the synthesis of MQD is driven by PEI-mediated electrostatic adsorption. When combined with antibody, the potential of immuno-SiO_2_-MQDs is significantly reduced (Figure 1F).

**Scheme 2:**
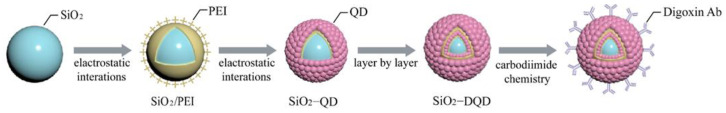
Synthesis of SiO_2_-MQDs nanocomposite.

The ICA for SARS-CoV-2 detection was designed and constructed by integrating four parts into a strip, including a sample pad, a test line coating the streptavidin, a control line coating goat anti-mouse IgG, and an absorbent pad (Scheme 2).

### 3.2. Optimization of RT-RPA and ICA

In order to shorten the experimental time and ensure accurate and readable experimental results, we have integrated the experimental steps and optimized the primer concentration and detection system.

**Figure 2:**
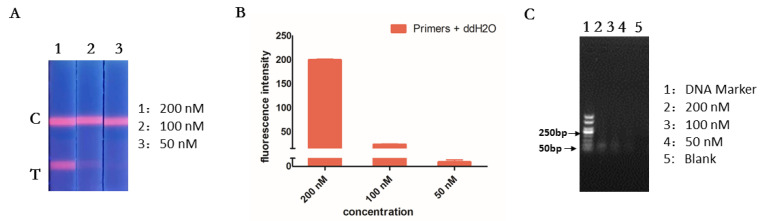
Specific primers screening without RNA template. (**A**) Primers with concentrations of 200 nM and 100 nM produced non-specific products, leading to false-positive results. (**B**) Corresponding fluorescence intensities of primers at different concentrations. Error bars represent the standard deviation of three repetitive experiments. (**C**) After the RPA product was subjected to agarose gel electrophoresis, the position was at 50 bp, far from the amplified product’s theoretical size (201 bp). Therefore, it is determined that the red band in (**A**) is a primer dimer. Strip 3 in (**A**) and line 4 in (**C**) represent primers at 50 nM, and no obvious non-specific amplification was observed.

**Figure 3:**
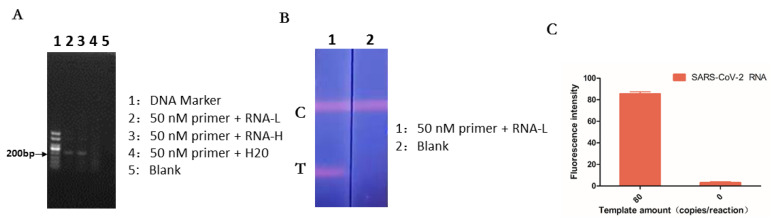
Specific primers screening with RNA template. (**A**) When the primer concentration was fixed at 50 nM, low-concentration RNA template (RNA-L) and high concentration RNA template (RNA-H) were added, respectively. Agarose gel electrophoresis shows that the size of the RPA product is consistent with the theoretical size (201 bp). (**B**) Immunochromatography was performed on the low-concentration product, and the result was positive. (**C**) Corresponding fluorescence intensities of RPA products and blank. Error bars represent the standard deviation of three repetitive experiments.

RPA primers are longer than conventional PCR primers and prone to mismatch forming dimers or secondary structures, resulting in non-specific amplification. Therefore, before the template reaction, it is necessary to perform non-specific verification of different concentrations of the primers. As shown in Figure 2, primers with concentrations of 200 and 100 nM produced non-specific products, leading to false-positive results. When the primer concentration was reduced to 50 nM, a low-concentration RNA template (RNA-L, 80 copies) and high concentration RNA template (RNA-H, 800 copies) were added, respectively. It can be seen from the agarose gel electrophoresis that the size of the RPA product is consistent with the theoretical size (201 bp), and the ICA result was positive (Figure 3A,B). Therefore, the forward and reverse primers concentrations were determined to be 50 nM for the SARS-CoV-2 assay.

Reverse transcription and RPA processes shorten the assay time. It is mentioned in the instructions for the reverse transcription kit and RPA kit that the reaction time of the two takes 60 and 30 min, respectively. We noticed that the temperature of the two reactions was about 40 °C, so we tried adding reverse transcriptase directly to the RPA system to synchronize reverse transcription and isothermal amplification. The experimental results in Figure 3A proved that the process was feasible and reliable.

**Figure 4:**
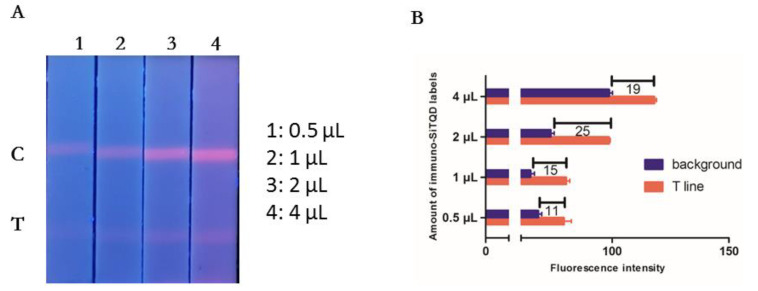
Screening of the amount of immuno-SiMQD labels. (**A**) Different amounts of MQD-labeled digoxin antibodies were used to compare the chromogenic effects. When the amount of template added was 8 copies and the primer concentration was 50 nM, the RPA products were used for testing. (**B**) When the added volume was 2 μL, the fluorescence intensity difference between the background and control was the most significant. Error bars represent the standard deviation of three repetitive experiments.

When optimizing the detection system, different amounts of MQD-labeled digoxin antibodies were used to compare the chromogenic effects. When the amount of template added was eight copies, and the primer concentration was 50 nM, the RPA products were used for testing. The added volume of labeled antibody in the sample loading system was 0.5, 1, 2, and 4 μL, respectively (Figure 4). When the added volume was 2 and 4 μL, the T line can be clearly seen, but because the background color of the 4 μL system is high, the volume of antibody added was determined to be 2 μL.

### 3.3. Specificity of the RT-RPA-Combined ICA

**Figure 5:**
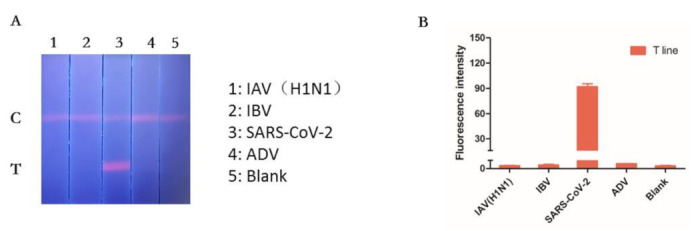
Specificity of the RT-RPA-combined ICA. (**A**) The RPA product of the SARS-CoV-2 shows a bright T line, while no positive results were seen for other respiratory viruses, which indicates that the designed system has excellent specificity for SARS-CoV-2. (**B**) Corresponding fluorescence intensities of the test lines of various respiratory viruses. Error bars represent the standard deviation of three repetitive experiments.

Since the clinical symptoms caused by SARS-CoV-2 and other respiratory viruses are similar [34], cross-reactivity with other viruses needs to be examined. Inactivated SARS-CoV-2, IAV, IBV, and ADV were adjusted to the same concentration of 10^3^ copies/μL and were amplified in accordance with the above process to verify the assay specificity. Each sample was independently repeated thrice, and the image was recorded and processed to calculate its signal value. As seen in Figure 5, the RPA product of the SARS-CoV-2 shows a bright T line, while other respiratory viruses do not show positive results. The result shows that the designed system has excellent specificity for SARS-CoV-2.

### 3.4. Sensitivity of the RT-RPA-Combined ICA

**Figure 6:**
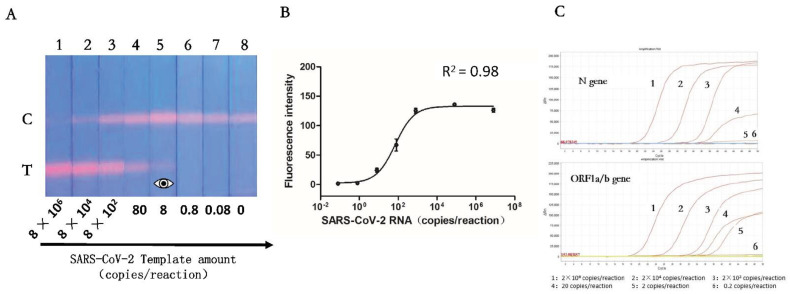
Sensitivity of the RT-RPA-combined ICA. (**A**) Images of test strips at different concentrations of SARS-CoV-2 RNA in the range of 0.08 to 8 × 10^6^ copies per reaction. (**B**) A four-parameter curve was fitted based on signal intensity and template concentration. Error bars represent the standard deviation of three repetitive experiments. (**C**) RT-qPCR results of *N* gene and *ORF1a/b* gene.

The sensitivity test shows the minimum detection limit of the method. After viral lysis, the released RNA templates of SARS-CoV-2 were accurately quantified by digital PCR (QIAGEN, Redwood City, CA, USA) with the concentration of 4 × 10^6^ copies/μL (see Supplementary Material S1). Then, RNA templates to be subjected to RT-RPA assay were serially diluted to simulate different concentrations of viral nucleic acid samples. As shown in Figure 6A, the red T line becomes weaker as the template concentration decreases. A four-parameter curve is fitted based on signal intensity and template concentration in Figure 6B. The IUPAC protocol (LOD = yblank + 3SDblank) was adopted to determine the limits of detection (LOD) of this assay, where yblank and SDblank are the average fluorescence intensity and standard deviation of the blanks, respectively [35,36,38]. Therefore, the LOD (before RPA amplification but not before immunodetection) for SARS-CoV-2 was estimated as two copies per reaction in this assay. The RT-qPCR kit (Daan Gene, Guangzhou, China) was used to detect the same SARS-CoV-2 RNA templates with an LOD of 20 copies for the *N* gene and 2 copies for the ORF1a/b gene per reaction in Figure 6C [37]. It can be seen that the detection scheme established in this experiment can achieve the same sensitivity as the commercial SARS-CoV-2 RT-qPCR kits. However, the scheme established in this study is fast, portable, and does not need to rely on instruments. It may have very important applications in limited scenarios, such as primary hospitals, underdeveloped areas, and self-examination at home.

### 3.5. Repeatability of the RT-RPA-Combined ICA

**Figure 7:**
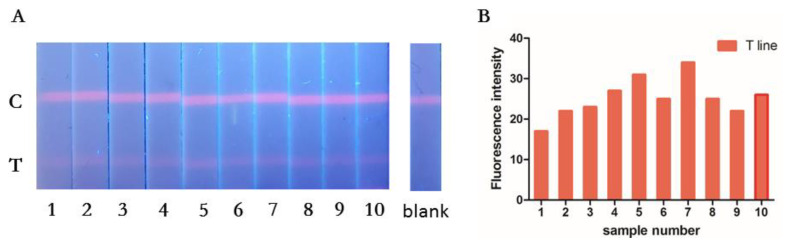
Repeatability of the RT-RPA-combined ICA. SARS-CoV-2 RNA templates were added to the reaction system for detection. (**A**) The detection was performed 10 times, and the detection rate was 100%. (**B**) Corresponding fluorescence intensities of SARS-CoV-2 RNA template.

In order to examine the repeatability of this protocol, 40 copies of the SARS-CoV-2 RNA template were used for detection. The detection was performed 10 times, and the detection rate was 100% (Figure 7). The fluorescence signal values of 10 tests were measured and counted, the average signal value was 25.2, and the relative standard deviation (RSD%) was 19%.

## 4. Conclusions

In this study, we have combined RPA nucleic acid amplification technology and multilayer quantum dot-based immunochromatography technology to develop a fast and convenient on-site detection solution for SARS-CoV-2. The scheme does not rely on instruments and can amplify viral nucleic acid with the body temperature (37 °C) of the operator. Compared with traditional nucleic acid extraction and detection technology, which takes at least 3 h, the whole process of this scheme can be completed within 1 h, with similar sensitivity. In addition, compared with RT-qPCR, the cost of RT-RPA is mainly the RPA kit (less than USD3 per reaction), and the instrument cost can be ignored. We believe that the scheme established in this study may provide a reference for rapid, portable, low-cost and sensitive detection of SARS-CoV-2 viruses.

## Data Availability

The datasets generated during and/or analyzed during the current study can be find in the main text and the supplementary materials.

## Supplementary Materials

The following supporting information can be downloaded at: https://www.mdpi.com/article/10.3390/pathogens11111252/s1, Figure S1: Raw results of Digital PCR; Figure S2: One-dimensional distribution map of each sample well of F1 channel (ORF1a/b gene); Figure S3: One-dimensional distribution map of each sample well of N channel (*N* gene); Figure S4: Two-dimensional distribution map of F1 channel (ORF1a/b) and N channel (*N* gene).

## References

[1] Yang X., Yu Y., Xu J., Shu H., Xia J., Liu H., Wu Y., Zhang L., Yu Z., Fang M. (2020). Clinical course and outcomes of critically ill patients with SARS-CoV-2 pneumonia in Wuhan, China: A single-centered, retrospective, observational study. Lancet Respir. Med..

[2] Wu F., Zhao S., Yu B., Chen Y.-M., Wang W., Song Z.-G., Hu Y., Tao Z.-W., Tian J.-H., Pei Y.-Y. (2020). A new coronavirus associated with human respiratory disease in China. Nature.

[3] Chan J.F.-W., Yuan S., Kok K.-H., To K.K.-W., Chu H., Yang J., Xing F., Liu J., Yip C.C.-Y., Poon R.W.-S. (2020). A familial cluster of pneumonia associated with the 2019 novel coronavirus indicating person-to-person transmission: A study of a family cluster. Lancet.

[4] Zhu N., Zhang D., Wang W., Li X., Yang B., Song J., Zhao X., Huang B., Shi W., Lu R. (2020). A Novel Coronavirus from Patients with Pneumonia in China, 2019. N. Engl. J. Med..

[5] Mohit E., Rostami Z., Vahidi H. (2021). A comparative review of immunoassays for COVID-19 detection. Expert Rev. Clin. Immunol..

[6] Yoo H., Kim I.-H., Kim S. (2021). Nucleic Acid Testing of SARS-CoV-2. Int. J. Mol. Sci..

[7] Dutta D., Naiyer S., Mansuri S., Soni N., Singh V., Bhat K.H., Singh N., Arora G., Mansuri M.S. (2022). COVID-19 Diagnosis: A Comprehensive Review of the RT-qPCR Method for Detection of SARS-CoV-2. Diagnostics.

[8] (2021). CDC 2019-Novel Coronavirus (2019-nCoV) Real-Time RT-PCR Diagnostic Panel.

[9] Abbasi H., Tabaraei A., Hosseini S.M., Khosravi A., Nikoo H.R. (2021). Real-time PCR Ct value in SARS-CoV-2 detection: RdRp or N gene?. Infection.

[10] Adams E.R., Ainsworth M., Anand R., Andersson M.I., Auckland K., Baillie J.K., Barnes E., Beer S., Bell J.I., Berry T. (2020). Antibody testing for COVID-19: A report from the National COVID Scientific Advisory Panel. Wellcome Open Res..

[11] Flower B., Brown J.C., Simmons B., Moshe M., Frise R., Penn R., Kugathasan R., Petersen C., Daunt A., Ashby D. (2020). Clinical and laboratory evaluation of SARS-CoV-2 lateral flow assays for use in a national COVID-19 seroprevalence survey. Thorax.

[12] Serrano M.M., Rodríguez D.N., Palop N.T., Arenas R.O., Córdoba M.M., Mochón M.D.O., Cardona C.G. (2020). Comparison of commercial lateral flow immunoassays and ELISA for SARS-CoV-2 antibody detection. J. Clin. Virol..

[13] Theel E.S., Slev P., Wheeler S., Couturier M.R., Wong S.J., Kadkhoda K. (2020). The Role of Antibody Testing for SARS-CoV-2: Is There One?. J. Clin. Microbiol..

[14] Liu H.-B., Du X.-J., Zang Y.-X., Li P., Wang S. (2017). SERS-Based Lateral Flow Strip Biosensor for Simultaneous Detection of Listeria monocytogenes and Salmonella enterica Serotype Enteritidis. J. Agric. Food Chem..

[15] Cha H., Kim H., Joung Y., Kang H., Moon J., Jang H., Park S., Kwon H.-J., Lee I.-C., Kim S. (2022). Surface-enhanced Raman scattering-based immunoassay for severe acute respiratory syndrome coronavirus 2. Biosens. Bioelectron..

[16] Hang Y., Boryczka J., Wu N. (2021). Visible-light and near-infrared fluorescence and surface-enhanced Raman scattering point-of-care sensing and bio-imaging: A review. Chem. Soc. Rev..

[17] Li H., Dong B., Dou L., Yu W., Yu X., Wen K., Ke Y., Shen J., Wang Z. (2020). Fluorescent lateral flow immunoassay for highly sensitive detection of eight anticoagulant rodenticides based on cadmium-free quantum dot-encapsulated nanospheres. Sensors Actuators B Chem..

[18] Rong Z., Wang Q., Sun N., Jia X., Wang K., Xiao R., Wang S. (2018). Smartphone-based fluorescent lateral flow immunoassay platform for highly sensitive point-of-care detection of Zika virus nonstructural protein 1. Anal. Chim. Acta.

[19] Shao Y., Duan H., Guo L., Leng Y., Lai W., Xiong Y. (2018). Quantum dot nanobead-based multiplexed immunochromatographic assay for simultaneous detection of aflatoxin B1 and zearalenone. Anal. Chim. Acta.

[20] Hu J., Zhang Z.-L., Wen C.-Y., Tang M., Wu L.-L., Liu C., Zhu L., Pang D.-W. (2016). Sensitive and Quantitative Detection of C-Reaction Protein Based on Immunofluorescent Nanospheres Coupled with Lateral Flow Test Strip. Anal. Chem..

[21] Li J., Lv Y., Li N., Wu R., Xing M., Shen H., Li L.S., Chen X. (2019). Robust synthesis of bright multiple quantum dot-embedded nanobeads and its application to quantitative immunoassay. Chem. Eng. J..

[22] Huang L., Liao T., Wang J., Ao L., Su W., Hu J. (2018). Brilliant pitaya-type silica colloids with central–radial and high-density quantum dots incorporation for ultrasensitive fluorescence immunoassays. Adv. Funct. Mater..

[23] Zheng S., Wu T., Li J., Qin J., Xiao R., Wang S., Wang C. (2022). Difunctional immunochromatographic assay based on magnetic quantum dot for ultrasensitive and simultaneous detection of multiple mycotoxins in foods. Sens. Actuators B Chem..

[24] Cheng X., Zheng S., Wang W., Han H., Yang X., Shen W., Wang C., Wang S. (2021). Synthesis of two-dimensional graphene oxide-fluorescent nanoprobe for ultrasensitive and multiplex immunochromatographic detection of respiratory bacteria. Chem. Eng. J..

[25] Wang C., Cheng X., Liu L., Zhang X., Yang X., Zheng S., Rong Z., Wang S. (2021). Ultrasensitive and Simultaneous Detection of Two Specific SARS-CoV-2 Antigens in Human Specimens Using Direct/Enrichment Dual-Mode Fluorescence Lateral Flow Immunoassay. ACS Appl. Mater. Interfaces.

[26] Wang C., Yang X., Gu B., Liu H., Zhou Z., Shi L., Cheng X., Wang S. (2020). Sensitive and simultaneous detection of SARS-CoV-2-specific IgM/IgG using lateral flow immunoassay based on dual-mode quantum dot nanobeads. Anal. Chem..

[27] Wang C., Xiao R., Wang S., Yang X., Bai Z., Li X., Rong Z., Shen B., Wang S. (2019). Magnetic quantum dot based lateral flow assay biosensor for multiplex and sensitive detection of protein toxins in food samples. Biosens. Bioelectron..

[28] Chen H., Wang Y., Wei H., Rong Z., Wang S. (2022). A rapid water bath PCR combined with lateral flow assay for the simultaneous detection of SARS-CoV-2 and influenza B virus. RSC Adv..

[29] Li J., Macdonald J., von Stetten F. (2018). Review: A comprehensive summary of a decade development of the recombinase polymerase amplification. Analyst.

[30] Lobato I.M., O’Sullivan C.K. (2018). Recombinase polymerase amplification: Basics, applications and recent advances. TrAC Trends Anal. Chem..

[31] Daher R.K., Stewart G., Boissinot M., Bergeron M.G. (2016). Recombinase Polymerase Amplification for Diagnostic Applications. Clin. Chem..

[32] Mota D.S., Guimaraes J.M., Gandarilla A.M.D., Filho J.C.B.S., Brito  W.R., Mariuba  L.A.M., Mota D.S. (2022). Recombinase polymerase amplification in the molecular diagnosis of microbiological targets and its applications. Can J. Microbiol..

[33] Wang C., Yang X., Zheng S., Cheng X., Xiao R., Li Q., Wang W., Liu X., Wang S. (2021). Development of an ultrasensitive fluorescent immunochromatographic assay based on multilayer quantum dot nanobead for simultaneous detection of SARS-CoV-2 antigen and influenza A virus. Sens. Actuators B Chem..

[34] Havasi A., Visan S., Cainap C., Cainap S.S., Mihaila A.A., Pop L.-A. (2022). Influenza A, Influenza B, and SARS-CoV-2 Similarities and Differences—A Focus on Diagnosis. Front. Microbiol..

[35] Wang X., Choi N., Cheng Z., Ko J., Chen L., Choo J. (2016). Simultaneous Detection of Dual Nucleic Acids Using a SERS-Based Lateral Flow Assay Biosensor. Anal. Chem..

[36] Zhang D., Huang L., Liu B., Ni H., Sun L., Su E., Chen H., Gu Z., Zhao X. (2018). Quantitative and ultrasensitive detection of multiplex cardiac biomarkers in lateral flow assay with core-shell SERS nanotags. Biosens. Bioelectron..

[37] WHO (2020). Clinical Management of Severe Acute Respiratory Infection when Novel Coronavirus (nCoV) Infection Is Suspected. Interim Guidance.

[38] Hu J., Jiang Y.-Z., Wu L.-L., Wu Z., Bi Y., Wong G., Qiu X., Chen J., Pang D.-W., Zhang Z.-L. (2017). Dual-Signal Readout Nanospheres for Rapid Point-of-Care Detection of Ebola Virus Glycoprotein. Anal. Chem..

