# Supplementary material for: Recombinase Polymerase Amplification Combined with Fluorescence Immunochromatography Assay for On-Site and Ultrasensitive Detection of SARS-CoV-2
Source: Pathogens. 2022 Oct 28;11(11):1252. doi: 10.3390/pathogens11111252 (PMC9692701; doi:10.3390/pathogens11111252)
Supplement: Supplementary file 1 [file pathogens-11-01252-s001.zip › pathogens-1935833-supplementary.pdf]

Supporting Information

S1 Quantification procedure of RNA templates of SARS-CoV-2 by using digital PCR.

In this work, RNA templates of SARS-CoV-2 were quantified using digital PCR by the following steps.

(1) Use a lysis reagent to lyse the virus to release the RNA template.

(2) The lysate was diluted 100 times as the mother solution, followed by 4 times of 2-fold dilution, and the 5 concentrations of the dilution were loaded and tested according to the kit instructions.

(3) According to the detection results, multiply the pre-dilution factor to obtain the RNA template concentration and take the average as the RNA template concentration in the RPA experiment.

Reaction Mixes

Reaction Mix Name	Target Name	Dye	Channel	IC	Reference
XG	N<br>F1	Cy5<br>FAM	Red<br>Green	-<br>-	-<br>-

Absolute Quantification (Imaging step 1)

	Sample/NTC/Control	Reaction Mix	Target	IC	Control Type	Concentration copies/µL	CI (95%)	Mean concentration copies/µL	CI (95%)	Partitions valid	positive	negative	Threshold
A3	XG	XG	F1<br>N	-<br>-	-<br>-	3882.5<br>3991.6	0.7%<br>0.7%	1546.3<br>1580.9	84.1%<br>84.6%	25302<br>25302	24315<br>24401	987<br>901	70.76<br>52.00
B3	XG	XG	F1<br>N	-<br>-	-<br>-	1953.0<br>1991.6	1.0%<br>1.0%	1546.3<br>1580.9	84.1%<br>84.6%	25445<br>25445	20041<br>20204	5404<br>5241	70.76<br>52.00
C3	XG	XG	F1<br>N	-<br>-	-<br>-	981.9<br>981.4	1.4%<br>1.4%	1546.3<br>1580.9	84.1%<br>84.6%	25432<br>25432	13592<br>13587	11840<br>11845	70.76<br>52.00
D3	XG	XG	F1<br>N	-<br>-	-<br>-	475.6<br>493.9	2.0%<br>2.0%	1546.3<br>1580.9	84.1%<br>84.6%	25447<br>25447	7824<br>8071	17623<br>17376	70.76<br>52.00
E3	XG	XG	F1<br>N	-<br>-	-<br>-	237.2<br>238.5	2.9%<br>2.9%	1546.3<br>1580.9	84.1%<br>84.6%	25421<br>25421	4244<br>4265	21177<br>21156	70.76<br>52.00
G3	NTC	XG	F1<br>N	-<br>-	-<br>-	0.100<br>0.000	168.6%<br>-	0.100<br>0.000	168.6%<br>-	25448<br>25448	2<br>0	25446<br>25448	70.76<br>52.00

Figure S1. Raw results of Digital PCR The amplification in each reaction system is a multiplex-PCR. There are two primer probes designed for the ORF1a/b gene (F1) and N gene (N) fragments, and the fluorescent reporter groups are FAM and CY5, respectively. Well A3 is the stock solution after 1000-fold dilution of the RNA template lysate. B3 is the solution of A3 after 2-fold dilution. C3 is the solution of B3 after 2-fold dilution. D3 is the solution of C3 after 2-fold dilution. E3 is the solution of D3 after 2-fold dilution. G3 is no template negative control. The initial RNA template concentrations quantified for ORF1a/b gene and N gene were 3.86×10^6 copies/μL and 3.93×10^6 copies/μL. The two concentrations are basically the same. For the convenience of calculation, 4×10^6 copies/μL. is used as the initial concentration of RNA template.

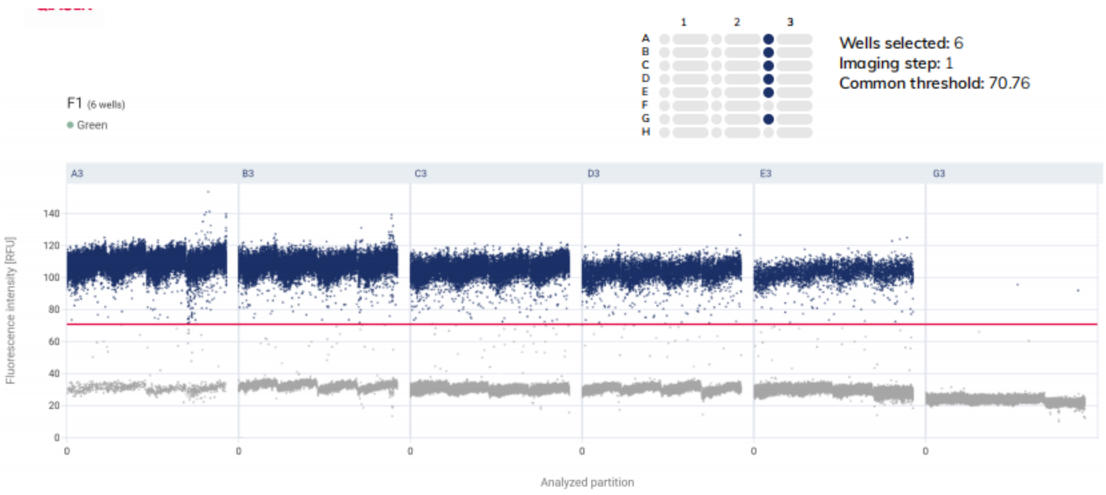

Figure S2. One-dimensional distribution map of each sample well of F1 channel (ORF1a/b gene).

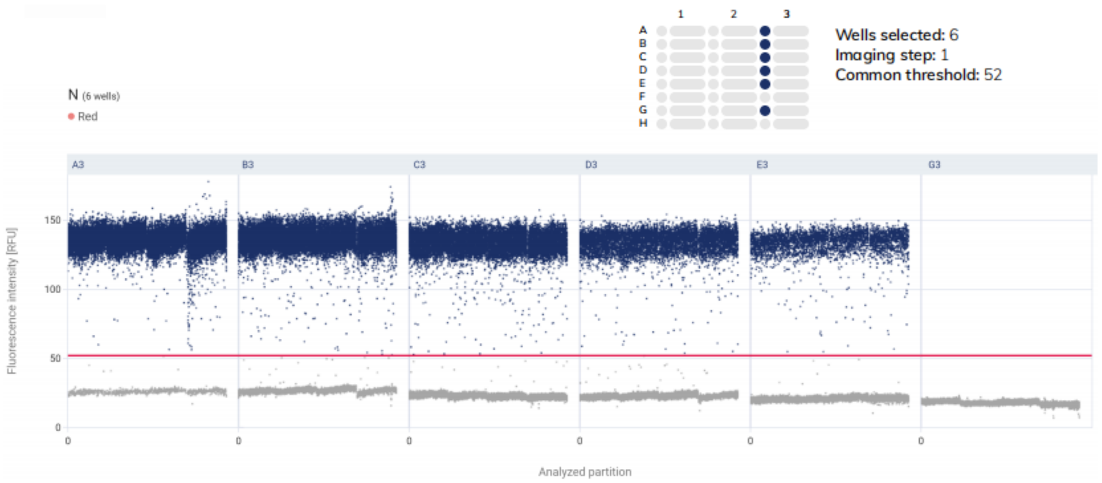

Figure S3. One-dimensional distribution map of each sample well of N channel (N gene).

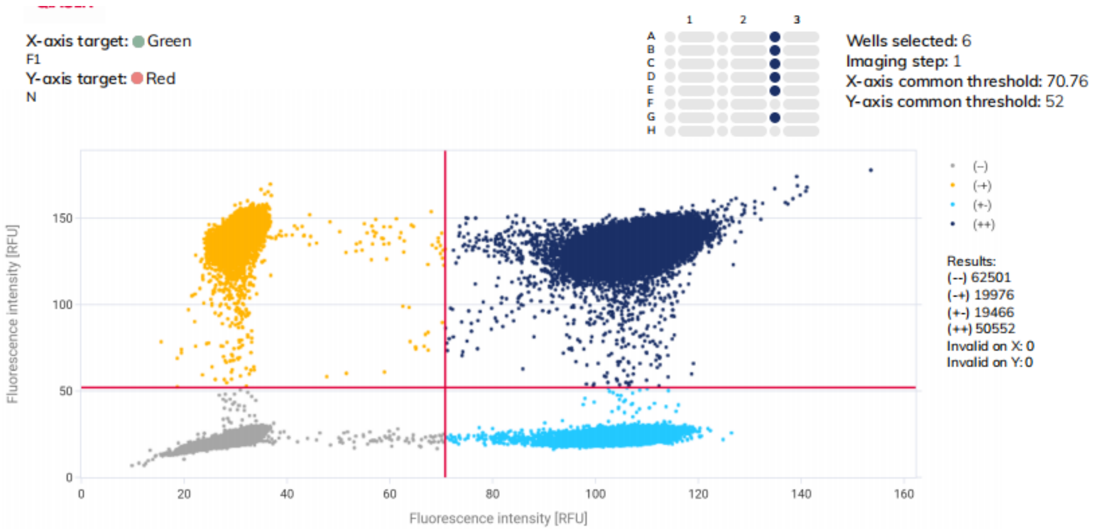

Figure S4. Two-dimensional distribution map of F1 channel (ORF1a/b) and N channel (N gene).
